# 4‐nitrobenzoate inhibits 4‐hydroxybenzoate polyprenyltransferase in malaria parasites and enhances atovaquone efficacy

**DOI:** 10.1002/1873-3468.70186

**Published:** 2025-10-14

**Authors:** Ignasi Bofill Verdaguer, Matheus Felipe Santos, Maurício Mazzine Filho, Gabriela Oliveira Castro, Agustín Hernández, Manoel Aparecido Peres, Alejandro Miguel Katzin, Marcell Crispim

**Affiliations:** ^1^ Department of Parasitology, Institute of Biomedical Sciences University of São Paulo Brazil; ^2^ Betternostics S.L. Noain Navarre Spain; ^3^ Department of Clinical and Toxicological Analysis, School of Pharmaceutical Sciences Federal University of Alfenas Brazil

**Keywords:** 4‐hydroxybenzoate, 4‐hydroxybenzoate polyprenyltransferase, 4‐nitrobenzoate, atovaquone, *COQ2*, malaria, *Plasmodium falciparum*, ubiquinone

## Abstract

Ubiquinone (UQ) is essential for the electron transport chain in *Plasmodium falciparum*, the causative agent of severe malaria. Its biosynthesis begins with the condensation of 4‐hydroxybenzoate (4‐HB) and an isoprenoid chain, catalyzed by 4‐HB polyprenyltransferase (4‐HPT; *COQ2* gene). Atovaquone (AV) inhibits the mitochondrial bc1 complex by competing with ubiquinol (UQH_2_), but resistance to the synergic combination AV/proguanil therapy has emerged. Here, we show that 4‐nitrobenzoate (4‐NB) inhibits *Pf*4‐HPT, enhances AV efficacy and selectivity, while preserving proguanil synergy. In *Saccharomyces cerevisiae* expressing *PfCOQ2*, 4‐NB inhibited UQ biosynthesis. *In vivo*, 4‐NB improved AV efficacy in *Plasmodium berghei*‐infected mice. Structure–activity studies with 4‐HB analogs further defined chemical features for potentiation. These findings support *PfCOQ2* as a target to boost AV‐based antimalarial therapy.

**Impact statement**

This study identifies a molecular rationale for enhancing atovaquone efficacy through targeted inhibition of ubiquinone biosynthesis. By validating *PfCOQ2* as a druggable target and demonstrating *in vivo* potentiation, our findings offer strategic advance toward rational antimalarial combination therapies, moving beyond empirical approaches and addressing current resistance.

## Graphical abstract

Atovaquone is an antimalarial requiring potentiation for sufficient efficacy. We pursued strategies to enhance its activity, showing that 4‐nitrobenzoate inhibits 4‐hydroxybenzoate polyprenyltransferase, decreasing ubiquinone biosynthesis. Since atovaquone competes with ubiquinol in mitochondria, 4‐nitrobenzoate facilitates its action, potentiating activity *in vitro* and *in vivo*. The figure illustrates drug potentiation (crossed rings), ubiquinone role (green), atovaquone (blue), and 4‐nitrobenzoate blocking 4‐hydroxybenzoate polyprenyltransferase (yellow).

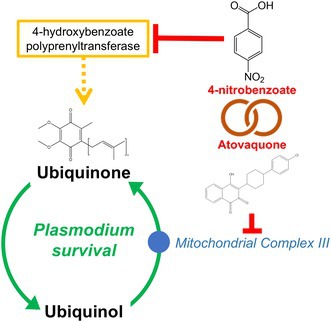

## Abbreviations

4‐BrB 4‐bromobenzoate

4‐ClB 4‐chlorobenzoate

4‐HB 4‐hydroxybenzoate

4‐HPT 4‐hydroxybenzoate polyprenyltransferase

4‐NB 4‐nitrobenzoate

AV Atovaquone

DHODH Dihydroorotate dehydrogenase

pABA para‐aminobenzoic acid

PAS para‐aminosalicylic acid

UQ Ubiquinone

UQH_2_ Ubiquinol

Malaria is one of the most widespread parasitic diseases in tropical and subtropical regions, affecting millions of people every year [1]. Most deaths occur in Africa due to *Plasmodium falciparum* infections. Several drug resistances have already been reported, and new antimalarial therapies are required [2, 3]. Atovaquone (AV) is one of the treatments for malaria. This compound competes with ubiquinol (UQH_2_) for binding to the cytochrome bc1 complex, thereby preventing ubiquinone (UQ) redox recycling [4, 5, 6]. As a consequence, the activity of dihydroorotate dehydrogenase (DHODH), involved in pyrimidine biosynthesis, is halted, leading to an inviable condition for the parasite. ATP production via oxidative phosphorylation does not seem to be essential in intraerythrocytic stages [7]. In the clinic, AV is synergistically combined with proguanil in a single formulation, Malarone^®^. Albeit a known inhibitor of folate biosynthesis, proguanil has been previously demonstrated to help AV to collapse the mitochondrial membrane potential, possibly by lowering the levels of ubiquinone; however, the precise mechanism by which it happens remains unknown [8]. The AV/proguanil combination is generally well tolerated in healthy adults using it for malaria prophylaxis or treatment [5]. In clinical trials and postmarketing experience, the most common adverse effects are mild gastrointestinal symptoms (nausea, vomiting, diarrhea, abdominal pain) and headache [5]. Major toxic effects are rare in practice; isolated case reports have noted severe cutaneous reactions (e.g., a 65‐year‐old developed Stevens–Johnson syndrome on prophylaxis) [9]. Rare hepatotoxic or hematologic events (e.g., liver injury or neutropenia) have been noted anecdotally, but causality is uncertain. Overall, the safety profile in otherwise healthy individuals is favorable: Common side effects are usually mild, and life‐threatening toxicities occur only rarely. In any case, parasitic resistance to AV plus proguanil has also been found, and new pharmacological combinations to potentiate AV are required [2, 10].

**Fig. 1:**
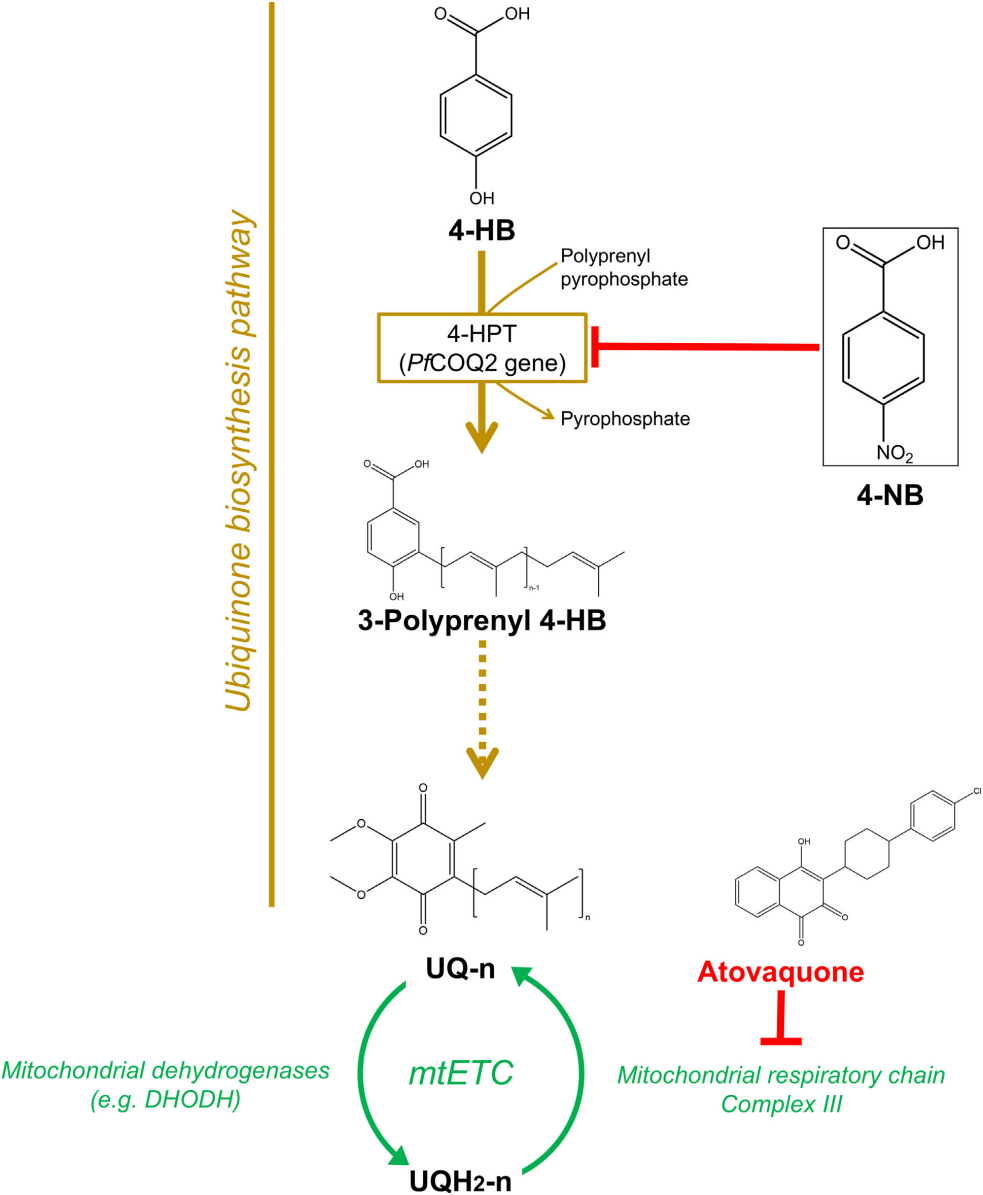
Ubiquinone biosynthesis pathway. Schematic representation of the ubiquinone biosynthetic pathway (brown), showing the chemical structures of precursors, intermediates, and UQ itself. The enzyme 4‐HPT (EC 2.5.1.39), the hypothetical target of 4‐NB in malaria parasites, is inside a brown box. Discontinuous lines represent multiple steps; red lines indicate drug inhibition. The chemical structure of 4‐NB is boxed, to indicate its inhibitor effect. The figure also shows UQ redox recycling in the mitochondrial electron transport chain (mtETC, green) and the chemical structure and molecular target of atovaquone. Abbreviations in the figure: Ubiquinone (UQ‐n) is the oxidized form of coenzyme Q with an isoprenoid side chain of length *n*, while ubiquinol (UQH2‐n) is its reduced form; *Pf*COQ2 refers to the *P. falciparum* 4‐hydroxybenzoate polyprenyltransferase enzyme; 4‐hydroxybenzoic acid (4‐HB) is a natural precursor of UQ, and 4‐nitrobenzoic acid (4‐NB) is a synthetic analog used as an inhibitor; dihydroorotate dehydrogenase (DHODH) is a key enzyme in the pyrimidine biosynthesis pathway involved in the mitochondrial electronic transport chain (mtETC).

*P. falciparum* biosynthesizes UQ‐8 and UQ‐9, while humans mostly produce UQ‐10 [11, 12, 13]. UQ biosynthesis occurs in the mitochondria and starts with the condensation of 4‐hydroxybenzoate (4‐HB) and an isoprenic chain by the transmembrane enzyme 4‐hydroxybenzoate polyprenyltransferase (4‐HPT), encoded by the *COQ2* gene (Fig. 1). The resulting compound, 3‐polyprenyl‐4‐hydroxybenzoate, is enzymatically modified by hydroxylations, decarboxylations, and three S‐adenosyl‐L‐methionine (SAM)‐mediated methylations, leading to UQ formation. In malaria parasites, 4‐HB for UQ aromatic head group biosynthesis is allegedly provided by the shikimate pathway and, possibly, exogenous incorporation [14]. On the other hand, the Methyl Erythritol Phosphate (MEP) pathway seems to be the source of isoprenoid precursors for UQ lateral chain formation [13]. The MEP pathway is located in a nonphotosynthetic plastid called the apicoplast, which leads to the production of two 5‐carbon molecules, isopentenyl pyrophosphate (IPP) and its isomer dimethylallyl pyrophosphate (DMAPP). Both substances can be subsequently condensed for the formation of longer polyprenyl moieties, including geranyl pyrophosphate (GPP, 10 carbons); farnesyl pyrophosphate (FPP, 15 carbons); geranylgeranyl pyrophosphate (GGPP, 20 carbons); and those directly employed for UQ biosynthesis, octaprenyl‐PP and nonaprenyl‐PP [13].

Previous studies in our group revealed that [^3^H] GGPP incorporation into UQ in malaria parasites can be inhibited by 4‐nitrobenzoate (4‐NB). Interestingly, although 4‐NB demonstrates poor antiplasmodial effects on its own, it has a significant ability to potentiate the efficacy of AV *in vitro*. Presumably, a depletion of the UQ pool would facilitate the AV interaction with mitochondrial complex III and thus diminish UQ‐redox regeneration required for DHODH activity. In any case, this potentiation effect is reduced when 4‐HB is added to the medium indicating that the target of 4‐NB is related to UQ biosynthesis [6]. In parallel to this, we also showed that the gene PF3D7_0607500 (*PfCOQ2*) can complement the *COQ2* gene of *S. cerevisiae*, demonstrating that *PfCOQ2* encodes for a functional 4‐HPT enzyme [15]. Consistent with these findings, previous studies revealed that the product of *PfCOQ2* is located in the parasitic mitochondria [16]. Despite all these findings, the enzymatic target of 4‐NB remained unknown and its utility for *in vivo* use unexplored.

In this study, we found that the molecular target of 4‐NB seems to be the 4‐HPT enzyme and that 4‐NB not only enhanced the *in vitro* antiplasmodial efficacy of AV but it also increased its selectivity compared to animal cells while still preserving the AV‐proguanil synergistic interaction. Additionally, 4‐NB boosted AV efficacy in treating murine malaria.

## Materials and methods

### Reagents, cells and stock solutions

[ring‐^14^C (U)] 4‐HB (50–60 mCi·mmol^−1^) was purchased from American Radiolabeled Chemicals (Saint Louis, Missouri, USA). FPP ammonium salt was purchased from Sigma‐Aldrich (code F6892). RPMI‐1640, medium, Albumax I (0.5%), and heat‐inactivated bovine fetal serum (BFS) were obtained from Thermo Fisher Scientific (Waltham, Massachusetts, USA). All HPLC grade solvents, AV, proguanil hydrochloride, and other reagents not cited here were purchased from Sigma (St. Louis, Missouri, USA). SYBR Green I^®^ nucleic acid gel stain from Life Technologies^®^ (Eugene, OR, USA), and UQ‐8,9 standards were purchased from Avanti Polar Lipids (Alabaster, Alabama, USA). Sterile stock solutions for *in vitro* use were prepared: 25 mM AV in dimethyl sulfoxide, filter‐sterilized 800 mM proguanil hydrochloride in water, and 4‐HB and all its analogs (including 4‐NB) were dissolved at 100 mM in ethanol. For *in vivo* use, the 4‐NB stock was prepared by dissolving the compound in PBS and adjusting the pH to 7.4 with sodium hydroxide until complete solubilization. Then, DMSO was added to a final concentration of 0.1%. The AV stock was prepared by dissolving 10 mg AV in 1 mL DMSO and then diluting it 1000‐fold in PBS for IP administration. All drugs used in mice were filter‐sterilized and stored at 4 °C. Erythrocytes (AB+) were a gift of Sírio Libanês Hospital (NESTA, São Paulo, Brazil). The monkey kidney epithelial cell line LLC‐MK2 (RRID:CVCL_3009) was obtained from the American Type Culture Collection (ATCC). All cell lines used in this study have been authenticated within the past three years by routine assessment of morphology, growth characteristics, and adhesion patterns. In addition, cultures were regularly tested and confirmed to be free of mycoplasma contamination. Animals (female BALB/c mice) were provided by the vivarium of the Institute of Biomedical Sciences and the vivarium of the School of Medicine of the University of São Paulo. Animals were housed in rooms with controlled temperature and humidity and a 12‐h light/dark cycle.

### Ethics statement

This study was carried out in strict accordance with the recommendations provided by the Guide for the Care and Use of Laboratory Animals of the Brazilian National Council of Animal Experimentation. The protocol and the study itself were approved by the Committee on the Ethics of Animal Experiments of the Institutional Animal Care and Use Committee at the Institute of Biomedical Sciences of the University of São Paulo (Protocol n° 2 420 280 823).

### *Plasmodium falciparum* asexual stages culture

The *P. falciparum* 3D7 strain, adapted to long‐term *in vitro* culture, was cultured in erythrocytes suspension in 75 cm^2^ flasks using RPMI‐1640 medium complemented with Albumax I (0.5%) and a gaseous mixture of 5% CO_2_, 5% O_2_, and 90% N_2_ obtained from Air Products Brasil LTDA (São Paulo, SP, Brazil) following the Trager and Jensen methodology [17, 18]. Culture synchronization was performed using a 5% (w/v) D‐sorbitol solution as previously described [19], and parasitic stages and parasitemia were monitored by Giemsa‐stained smears microscopy. To avoid culture contamination, PCR tests for mycoplasma were regularly carried out [20].

### Culture and complementation of *COQ2* gene in yeasts

The culture and introduction of *COQ2* genes in yeast were performed as previously described [15]. Cells were cultured in YPD medium (2% glucose, 2% peptone, 1% yeast extract), YPGly medium (2% glycerol, 2% peptone, 1% yeast extract), or Synthetic Defined medium (SD) without uracil and with 2% glucose or 3% glycerol as a carbon source. The *COQ2Δ S. cerevisiae* strain (YNR041C, MATa *ade2‐1 his3‐1,15 leu2‐3,1 12 trp1‐1 ura3‐1 COQ2:HIS3*) was generously provided by Dr. M. H. de Barros (Dept. Microbiology, University of São Paulo). The COQ2Δ yeast strain was complemented either with its endogenous gene or with the PF3D7_0607500 gene (codon‐optimized for yeast expression by GenScript), cloned into the p416‐GPD yeast expression vector and introduced into yeast cells using the lithium acetate transformation method [21]. *COQ2Δ* yeast complemented with the empty vector was also created. The phenotype was evaluated as the ability to use nonfermentable (glycerol) or fermentable (glucose) carbon sources [15].

### Yeast growth tests

Drop growth tests were conducted on agar plates with SD medium without uracil, using 2% glucose or glycerol as the carbon source, and with or without the addition of 1 mM 4‐HB, 1 mM 4‐NB, or a combination of 1 mM 4‐HB and 1 mM 4‐NB. Both 4‐HB and 4‐NB were added from a 100‐fold concentrated stock solution prepared in ethanol. For drop tests, cells from each strain were grown to early stationary phase in SC medium without uracil and with 2% glucose. The culture absorbance was then adjusted to 0.4 (approximately 4 × 10^6^ cells·mL^−1^) using sterile water, and several 10‐fold serial dilutions in water were prepared from the initial culture. Aliquots of 2.5 μL from each dilution were placed sequentially onto appropriate agar Petri dishes. The inoculated plates were then incubated at 30 °C for 48–72 h before being photographed.

### 4‐hydroxybenzoate polyprenyltransferase activity

The *in vitro* function of 4‐HPT was characterized using radioisotope assays in crude membrane extracts from the yeast *COQ2Δ* strain transformed with p416‐GPD‐PfCOQ2. For this, we choose not to use radiolabeled prenyl diphosphate as a marker because these intermediates are involved in multiple metabolic pathways in yeast, which could result in background signals and reduce the specificity of the assay. On the other hand, as far as we know, 4‐HB is exclusively used for ubiquinone biosynthesis in all organisms studied to date, which makes it a more selective and reliable substrate for tracking 4‐HPT activity. Yeasts were cultured to early stationary phase in SD plus glucose medium, and then, cells were disrupted by glass beads (0.5 mM diameter) [22]. Unbroken cells were removed by centrifugation at 900 × ***g*** for 1 min, and protein was adjusted to 50 mg·mL^−1^ with 100 mM Tris/HCl pH 7.5. Commercially available FPP was used as an isoprenic donor, as most 4‐HPT enzymes studied show broad substrate specificity for the isoprenic chain length [23, 24]. The reaction was performed in 1.5 mL microcentrifuge tubes and initiated by adding 4 mg of yeast protein to 92 μL of reaction buffer. The final concentrations in the reaction were adjusted to 10 mM MgCl_2_, 50 μm FPP, and 10 μm [ring‐^14^C(U)] 4‐HB in 100 mM Tris/HCl pH 7.5, along with drugs when applicable. All substances dissolved in ethanol—namely, the drugs and [ring‐^14^C(U)] 4‐HB—were dried under vacuum prior to use to prevent the introduction of organic solvents into the reaction. The remaining components were added subsequently. The volume was adjusted to 100 μL with 100 mM Tris/HCl pH 7.5, and the reaction was initiated by adding the yeast extract. In some assays, 4‐NB or sulfanilamide analogs were also added to the reaction, prenyl‐PP was omitted, or boiled yeast extracts were employed as controls. After 1 h of incubation at 37 °C, the reaction was stopped by adding 200 μL of ethyl acetate. The mixture was vortexed, centrifuged at 12000 × ***g*** for 10 min, and the organic phase was dried under vacuum. The residue was suspended in 10 μL of ethyl acetate and chromatographed on silica 60 plates (20 × 20 cm, Merck). Plates were developed with acetone: petroleum ether (7 : 3, by volume) [25]. Authentic standards of 4‐HB and p‐aminobenzoic acid (pABA) were run on the same plate. Standards were visualized with iodine vapor. Finally, the plates were treated with EN³HANCE® Autoradiography Enhancer (PerkinElmer®, Waltham, MA, USA) and exposed to autoradiography for one week at −70 °C. Products of the reaction were identified by their Rf and the controls previously described [25].

### Parasitic growth monitoring

The antimalarial effects of the compounds, separately or in combination, on parasitic growth were monitored relative to an untreated control using previously published methods [26, 27]. The experiments were done in 96‐well plates starting at the ring stage (2% parasitemia, 2% hematocrit). Several concentrations of each compound were prepared by serial dilution in RPMI complete medium. Parasite growth was monitored after 48 h by SYBR Green I^®^ DNA staining as described elsewhere [27]. Briefly, 100 μL of culture was incubated in a 96‐well cell plate in the darkness and at room temperature after adding 100 μL of SYBR Green I^®^ 1/5000 (v/v) in lysis buffer [20 mM Tris, pH 7.5; 5 mM EDTA; 0.008% saponin (w/v); 0.08% Triton X‐100 (v/v)]. Fluorescence was measured in a POLARstar Omega fluorometer^®^ (BMG Labtech^®^, Ortenberg, Germany) with excitation and emission bands centered at wavelengths of 485 and 520 nm, respectively.

### Assessment of antiplasmodial drug effects and drug interaction studies

The concentration of each compound required to decrease parasitic growth by 50% (IC_50_) was determined at 48 h. Inhibition of parasite growth was analyzed in relation to the logarithm of the concentration using a nonlinear regression (dose–response slope/variable sigmoid equation) with graphpad prism ^®^ software. All experiments monitoring parasitic growth were performed at least three times with four technical replicates for each one. For the study of AV interaction with compounds with poor antimalarial activity it was employed a potentiation assay, as described elsewhere [6]. In this case, the AV IC_50_ value was calculated at a fixed nontoxic concentration of different compounds, as determined in preliminary experiments. The single‐drug fractional inhibitory concentration value (FIC value) was calculated as the IC_50_ value of AV in a combined solution divided by the IC_50_ value of AV alone. AV FIC < 0.5 was considered indicative of a drug potentiation phenomenon [6].

### *In vivo* experiments

Three female BALB/c mice, aged 4–6 weeks, were used for each group, and experiments were performed three times. Mice were infected by intraperitoneal injection with 1 × 10^7^ *P. berghei*‐infected erythrocytes (the rodent malaria parasite), obtained from a donor mouse. The animals were kept in standard conditions with a 12‐h light/dark cycle, in cages with autoclaved pine wood and free access to water and food. On the fourth day postinfection, administration of 4‐NB or AV at subtherapeutic doses was initiated. 4‐NB was administered at 88 mg·kg^−1^ from a 5.3 g·L^−1^ PBS sterile stock solution. The chosen dose of 4‐NB corresponds to one‐tenth of the lethal dose 50 [28]. AV was administered at 0.1 mg·kg^−1^ from a 20 μg·mL^−1^ sterile stock solution [29]. For combinatory treatments, AV dissolved in DMSO was diluted in the previously described 4‐NB stock solution. In all cases, the drugs were administered once daily, every 24 h, over a period of 5 days. Parasitemia was monitored through tail blood smears every two days, and mice were euthanized when it exceeded 40%. To compare the means of variables, the unpaired Student's *t*‐test was employed. In addition, survival curves were plotted using the Kaplan–Meier method, and statistical differences were evaluated using the log‐rank test. The analyses were performed using the graphpad prism ^®^ 5.3 program.

### Animal cells culture and growth monitoring

The kidney epithelial cells from *Macaca mulatta* (LLC‐MK2) were grown routinely in 75 flasks in RPMI medium supplemented with 10% FBS and 10 mg·L^−1^ gentamicin sulfate. The cultures were maintained in a humidified incubator with 5% CO_2_ at 37 °C. Cells were manipulated following the passage and trypsinization procedures as described elsewhere [30, 31]. For experiments, confluent cultures were washed in phosphate‐buffered saline (PBS), trypsinized, centrifuged at 300 × ***g***, and suspended in culture media. The cells were cultured in 96‐well plates at a density of 1.0 × 10^5^ cells per well. The next day, the cells were washed with PBS and subjected to pharmacological treatments. Ethanol controls were included to take into consideration any effects related to solvents, and its concentration was always ≤ 1%. After 48 h, the cells were washed once in PBS, and each well was incubated at 37 °C with 50 μL PBS containing 5 mg·mL^−1^ 3‐(4,5‐Dimethyl‐2‐thiazolyl)‐2,5‐diphenyl‐2H‐tetrazolium bromide (MTT). After 4 h, 50 μL of 20% SDS in PBS (wt/vol.) was added to each well. The next day, the absorbance at 595 nm wavelength corrected to 690 nm was monitored in a POLARstar Omega fluorometer^®^ (BMG Labtech^®^, Ortenberg, Germany), and the results were analyzed by GraphPad Prism^®^ software to determine the 50% cytotoxic concentration (CC_50_) [30]. Statistical significance was determined by Student's *t*‐test, one‐way ANOVA, or nonlinear regression (dose–response). All experiments were repeated at least three times, with four technical replicates each time, and PCR for mycoplasma and optical microscopy were used to avoid culture contamination [32]. Selectivity Index (SI) was calculated as CC_50_ in cells/IC_50_ in parasites. As other authors have described, a selectivity index > 10 indicates promising antibiotic potential [33].

## Results

### Enhancement of atovaquone and proguanil activity by 4‐hydroxybenzoate analogs

**Fig. 2:**
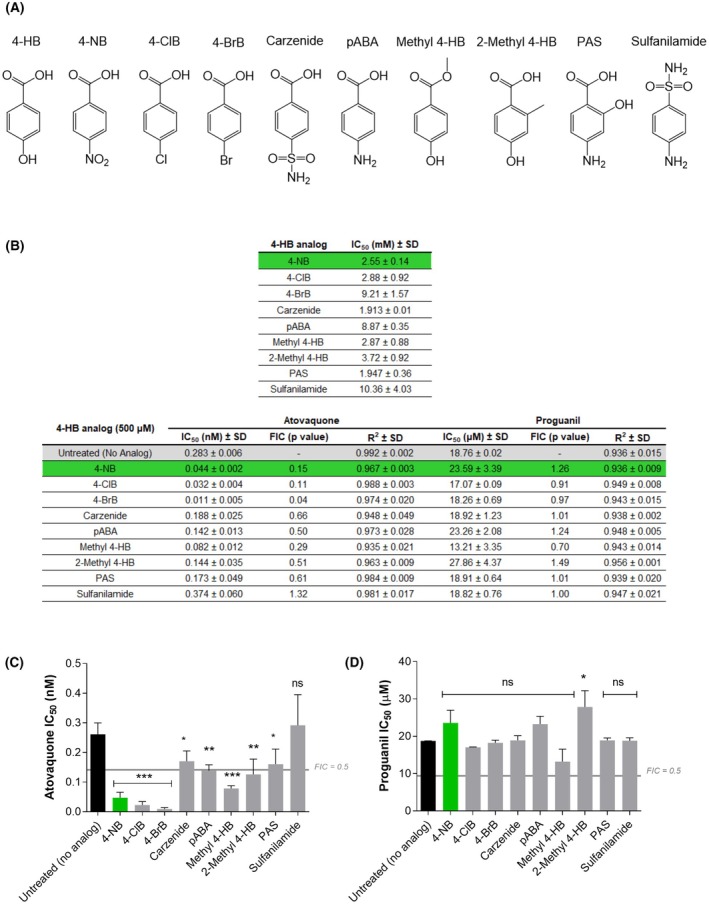
4‐Hydroxybenzoate analogs effects on atovaquone and proguanil activity. (A) Structures of each of the 4‐HB analog tested. (B) Values of the IC_50_ ± SD and *R* ^2^ values for each 4‐HB analog, as well as IC_50_ values of AV and Proguanil in routine medium or medium supplemented with each compound, as indicated. Results represent the mean of three independent experiments. Green highlights 4‐NB. (C and D) Panels C and D display the results shown in Table B for AV and Proguanil, respectively. Results were analyzed using one‐way analysis of variance (ANOVA) with Dunnett's multiple comparison test compared to the control. *P < 0.05; **P < 0.01; ***P < 0.001. Error bars indicate standard deviation (SD). The gray horizontal bar indicates FIC = 0.5, which corresponds to the threshold below which the effect is considered potentiation. Abbreviations: 4‐HB (4‐hydroxybenzoic acid), 4‐NB (4‐nitrobenzoic acid), 4‐ClB (4‐chlorobenzoic acid), 4‐BrB (4‐bromobenzoic acid), pABA (para‐aminobenzoic acid), PAS (para‐aminosalicylic acid), ns (statistically not significant), FIC (Fractional Inhibitory Concentration Index, which corresponds to the IC_50_ value of atovaquone in medium supplemented with each 4‐HB analog divided by the IC_50_ value of atovaquone alone).

The previous results regarding the potentiation of AV efficacy by 4‐NB prompted our interest in identifying the chemical structural features of molecules that could underlie this effect on AV activity. We tackled this by using other 4‐HB analogs. The first step was to create a collection of compounds to be explored (Fig. 2A). This collection included 4‐HB analogs with modifications to the substituent bound to C4, since these have previously shown inhibitory activity in UQ biosynthesis in other organisms [34]: 4‐NB, 4‐Bromobenzoate (4‐Chlorobenzoate), carzenide (4‐Sulfamoylbenzoic acid), and p‐aminobenzoic acid (pABA). Additionally, we included another analog with similar characteristics, 4‐Bromobenzoate (4‐BrB) or with radicals introduced in other positions of the phenyl ring: methyl 4‐hydroxybenzoate, para‐aminosalicylic acid (PAS), 2‐methyl 4‐hydroxybenzoate, and 2‐methyl hydroxybenzoate. Finally, as a control, we selected sulfanilamide (4‐aminobenzenesulfonamide), a structurally related compound to 4‐NB with no reported inhibitory activity against 4‐HPT enzymes. The IC_50_ results at 48 h (Fig. 2B) revealed low antiplasmodial activity for all compounds, ranging from 1 to 10 mM. For sulfanilamide, the observed antiplasmodial activity was extremely low, and the *R* ^2^ values did not indicate a clear dose–response relationship. Slightly higher antiplasmodial activity was observed for 4‐HB analogs with substitutions to the substituent in the C4 position. Within this group, it is found carzenide (IC_50_ = 1.913 ± 0.01 mM), followed by 4‐NB (IC_50_ = 2.55 ± 0.14 mM), 4‐ClB (IC_50_ = 2.88 ± 0.92 mM), and 4‐BrB (IC_50_ = 9.21 ± 1.57 mM). A review of the literature revealed that two compounds in the collection had been previously tested in the parasite: PAS [35] and sulfanilamide [36]. PAS has been described as a potent inhibitor of exogenous pABA transport (IC_50_ for transport of approximately 200 nm).

We then conducted pharmacological combination assays with Proguanil and AV. To this end, the IC_50_ values for Proguanil and AV in the presence of 0.5 mM of each 4‐HB analog (Fig. 2B–D) were calculated. A concentration of 0.5 mM was chosen because none of the compounds in our collection caused any effect on parasite proliferation after 48 h at that concentration. We observed pharmacological potentiation phenomena (individual FIC < 0.5) of AV using methyl 4‐HB, 2‐methyl 4‐HB, and all 4‐HB analogs with modifications to the substituent in the C4 position, except for carzenide. The compounds that showed the greatest potentiation effects on AV activity were 4‐NB, 4‐ClB, and 4‐BrB (FICs ranging from 0.05 to 0.15). No potentiation phenomena were observed for any antimalarial when using sulfanilamide (control), and only a reduction of approximately 30% in the IC_50_ value of Proguanil was achieved with methyl 4‐HB. We also observed that, overall, the most significant potentiation effects on AV activity were observed for 4‐HB analogs containing a small substituent (‐Br, ‐Cl, ‐NO_2_) at the C4 position. Additionally, methyl 4‐HB and 2‐methyl 4‐HB also showed some potential to enhance the effects of AV.

### 4‐hydroxybenzoate analogs inhibit recombinant *PfCOQ2*

**Fig. 3:**
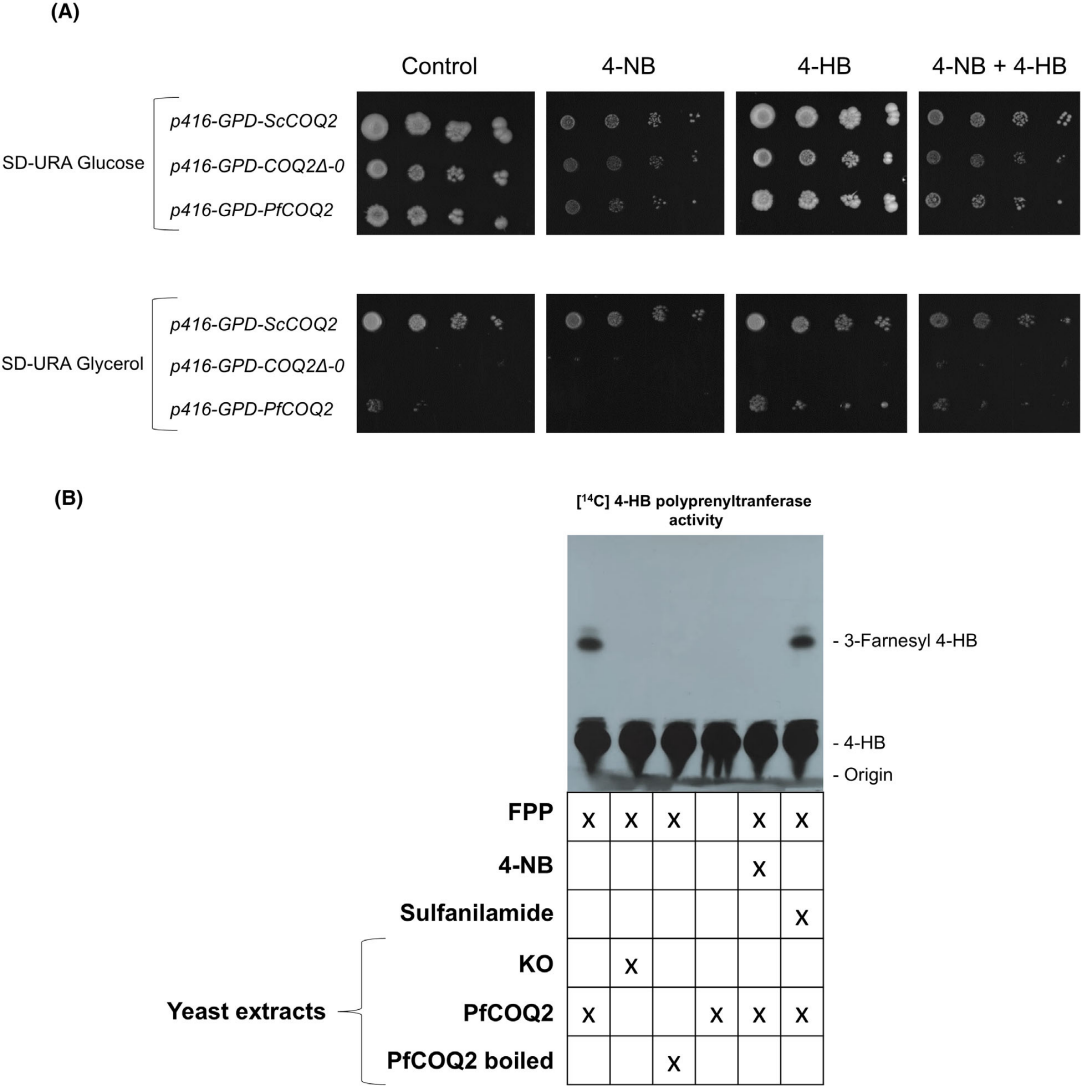
*PfCOQ2* complementation and enzymatic activity. (A) The figure shows the growth of yeast strains in SD + glucose/SD + glycerol media containing a concentration of 1 mM of different drugs, as indicated. This experiment was performed three times with similar results. (B) The figure shows the analysis of 4‐HPT enzymatic activity in *PfCOQ2‐complemented* yeasts in the presence of 0.5 mM of different drugs. The compounds added to the enzymatic reaction are indicated. Rf of 4‐HB and 3‐Farnesyl 4‐HB are also indicated. This experiment was performed three times with similar results. Abbreviations: 4‐NB (4‐nitrobenzoic acid), 4‐HB (4‐hydroxybenzoic acid), SD‐URA (synthetic defined medium without uracil), p416‐GPD vector (yeast expression plasmid carrying the constitutive GPD promoter, from the *S. cerevisiae* glyceraldehyde‐3‐phosphate dehydrogenase gene), COQ2 (4‐hydroxybenzoate polyprenyltransferase), *Sc*COQ2 (*Saccharomyces cerevisiae* COQ2 gene), *Pf*COQ2 (*P. falciparum* COQ2 gene), COQ2Δ‐0 (yeast strain with complete deletion of the COQ2 gene), FPP (farnesyl pyrophosphate), KO (knockout).

Despite the last results, we decided to further focus on 4‐NB because previous studies showing its detrimental effects on UQ biosynthesis in malaria parasites [6]. As the enzymatic target of 4‐NB in *P. falciparum* remains unknown, we hypothesized that it could be the 4‐HPT enzyme. To assess this, a set of yeast strains was created to study the pharmacological inhibition of the malaria parasite 4‐HPT enzyme. The *COQ*2Δ strain was transformed with either the p416‐GPD empty plasmid (referred to as the p416‐GPD‐*COQ*2Δ‐0 strain), the p416‐GPD‐ScCOQ2 plasmid (referred to as the Sc*COQ*2 strain), or the p416‐GPD‐Pf*COQ*2 plasmid (referred to as the *PfCOQ*2 strain). The phenotype of these strains was assessed by their ability to use a nonfermentable carbon source (SD + glycerol) in the presence or absence of different combinations of 4‐HB and 4‐NB (Fig. 3A; see another replicate in Supporting Information, Fig. S1). As expected, the *COQ*2Δ − 0 strain was only able to grow on SD + glucose plates, while all strains expressing *COQ*2 genes were able to grow on both SD + glucose and SD + glycerol plates. The addition of 1 mM 4‐NB negatively affected the growth of all strains in SD + glucose medium. In SD + glycerol, 4‐NB completely inhibited the growth of the *p416‐GPD‐PfCOQ2* strain but not the *p416‐GPD‐ScCOQ2* strain. This may indicate that 4‐NB has a mechanism of action related to respiratory metabolism and directly involves the 4‐HPT enzyme of malaria parasites. The more pronounced effects observed in yeast expressing the parasite enzyme, compared to those expressing the endogenous yeast enzyme, may indicate that the latter is either less sensitive to the inhibitor, more highly expressed, or has greater functional activity. As a result, inhibition of its enzymatic function and the subsequent impairment of mitochondrial activity are less susceptible to 4‐HPT enzyme inhibitors. The addition of 1 mM 4‐HB to both media partially rescued the growth of the *p416‐GPD‐PfCOQ2* strain from the effects of 4‐NB on SD + glycerol plates, indicating that 4‐NB acts as an antimetabolite of 4‐HB. 4‐HB itself had no detrimental effect on yeast strains' growth in either SD + glucose or SD + glycerol media. In fact, we observed that the addition of 4‐HB leads to better growth in the *p416‐GPD‐PfCOQ2* enzyme compared to control cells. One possible explanation is that the Plasmodium enzyme may be less efficient than the endogenous yeast enzyme, and this limitation could be partially overcome by increasing substrate availability. Supplementation with 4‐HB might therefore enhance ubiquinone biosynthesis or reduce the cellular energy cost of 4‐HB production.

Considering the last set of results, we wanted to directly assess the enzymatic inhibition of *p416‐GPD‐PfCOQ2* (Fig. 3B; see another replicate in Supporting Information, Fig. S1). When whole yeast extracts were incubated with [ring‐^14^C (U)] 4‐HB plus FPP, radiolabeled compounds were produced which were chromatographically compatible with 3‐farnesyl 4‐HB (Fig. 3B), as previously described [25]. Other radiolabeled compounds were also observed, with one of them being identified as a nonenzymatically produced contaminant derived from [ring‐^14^C (U)] 4‐HB (It is observed a flattened spot just above the radioactive 4‐HB on the TLC, which was also present when using heat‐inactivated extracts. This indicates that the origin of the spot is nonenzymatic). The other compounds may be SAM‐methylated derivatives of 3‐polyprenyl 4‐HB. This type of compound formation is commonly seen when assaying the product of *COQ2* activity in raw extracts [24]. However, prenylated derivatives of [ring‐^14^C (U)] 4‐HB were not observed in *PfCOQ2* boiled extracts, extracts of *COQ2Δ‐0* strain, or assays with *PfCOQ2* strain without the addition of polyprenyl‐PP, which indicates the enzymatic origin of these compounds as well as their prenylated nature. Furthermore, the addition of 0.5 mM 4‐NB inhibited the prenylation of [ring‐^14^C (U)] 4‐HB. Similarly, the aromatic compound sulfanilamide did not inhibit this 4‐HPT activity. Sulfanilamide acts as a competitive inhibitor of the bacterial enzyme dihydropteroate synthetase, specifically competing with p‐aminobenzoic acid (pABA), a compound structurally similar to 4‐hydroxybenzoate (4‐HB). In our study, sulfanilamide was used as a control to demonstrate that not all compounds with similar chemical structures are capable of inhibiting 4‐HB utilization by the 4‐HPT enzyme. Furthermore, it is worth noting that since sulfanilamide does not inhibit 4‐HPT, it also does not potentiate the activity of atovaquone. Finally, we would like to make a few considerations regarding our methodological approaches. First, FPP was used as the prenyl donor in our assays because it is commercially available, cost‐effective, and easier to handle compared with natural long‐chain substrates such as octaprenyl‐ or nonaprenyl‐pyrophosphate.

Moreover, previous studies have shown that several 4‐HPT enzymes display relaxed specificity for prenyl chain length, efficiently accepting shorter‐chain isoprenoids like FPP. It should be noted that this experiment indicates that the 4‐HPT enzyme of *P. falciparum*—as proven by yeast complementation assays—produces a substance derived from 4‐HB only when FPP is added. Furthermore, this substance exhibits a chromatographic retention time consistent with farnesyl 4‐HB, as reported in studies performing similar enzymatic assays [25]. While we acknowledge that definitive structural confirmation of the enzymatic product (e.g., by LC–MS/MS or NMR) would provide the ultimate validation, our combined evidence from yeast complementation, substrate rescue experiments, and radiolabeled assays strongly supports the conclusion that *PfCOQ2* encodes a functional 4‐HPT inhibited by 4‐NB. Future studies should pursue a more detailed enzymatic characterization, including structural identification of the reaction products, to deepen this biochemical evidence.

### 4‐nitrobenzoate enhances AV selectivity, potency, and synergy with proguanil

Since both proguanil and 4‐NB seem to inhibit UQ biosynthesis in malaria parasites, the effects of 4‐NB on proguanil were investigated (Table 1). The IC_50_ values of AV and proguanil were calculated as a reference for further comparison. Then, the same assay was performed by supplementing the RPMI medium with 0.5 mM 4‐NB, a concentration which has no effect on parasitic growth at 48 h, as described elsewhere [6]. Results showed that 4‐NB potentiates AV efficacy × 6.25‐fold but not proguanil. These results demonstrate that 4‐NB enhances the antiplasmodial efficacy of AV against parasites while maintaining the efficacy of proguanil. This suggests that the mechanisms by which proguanil and 4‐NB potentiate AV are likely different.

**Table 1:** 4‐NB effects on AV potency and selectively. The table shows the IC_50_/CC_50_ (average ± SD) values for proguanil and AV, as well as the SI values for AV, in the absence/presence of 0.5 mM 4‐NB in *P. falciparum* parasites and LLC‐MK2 cells. This experiment was performed three times.

	IC_50_ proguanil in malaria parasites (μm)	IC_50_ atovaquone in malaria parasites (nm)	CC_50_ atovaquone in LLC‐MK2 cells (μm)	SI atovaquone
Control	18.76 ± 0.02	0.550 ± 0.012	5.105 ± 1.520	0.928 × 10^4^
4‐NB	23.59 ± 3.39	0.088 ± 0.004	3.817 ± 2.012	4.34 × 10^4^

Other authors demonstrated that AV also inhibited cytochrome bc1 and mtETC in mammalian cells [37]. Thus, it was decided to evaluate how a potential combination of AV with 4‐NB would affect the selectivity of the antimalarial. The presence of a 0.5 mM concentration of 4‐NB slightly reduced the CC_50_ value of AV in the LLC‐MK2 cell line (Table 1). However, statistical analysis showed that the observed potentiating effect was not significant.

### 4‐nitrobenzoate enhances atovaquone efficacy *in vivo*

**Fig. 4:**
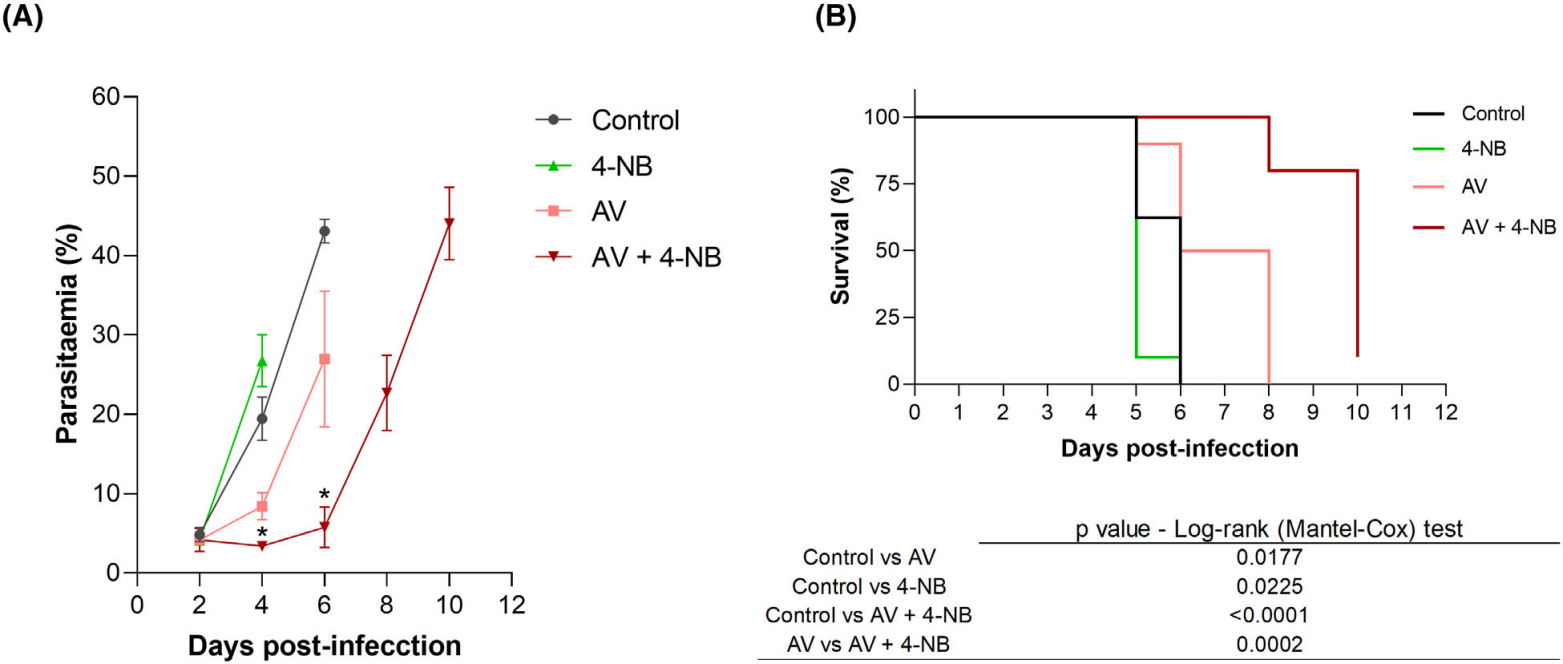
4‐Nitrobenzoate improves the efficacy of atovaquone *in vivo*. (A) Parasitemia in *P. berghei* ANKA‐infected mice treated with atovaquone (AV), 4‐nitrobenzoate (4‐NB), or their combination. All groups received the same amount of vehicle. Data are from one representative experiment with three animals per group (see additional data in Supporting Information, Fig. S2). (B) Survival of *P. berghei*‐infected mice treated as in (A). The curves represent the mean of two identical experiments, each with three animals per group (see table for statistical details). Statistical analyses were performed using unpaired Student's t‐test (parasitemia) and log‐rank (Mantel‐Cox) test (survival); *P < 0.05. Error bars indicate Standard Error of Mean (SEM). If not indicated, no significant differences were observed. Abbreviations: 4‐NB (4‐nitrobenzoic acid), AV (atovaquone).

As observed, 4‐NB enhanced the selectivity and potency of AV. Therefore, it was of interest to test this compound in mice, either alone or in combination with AV. For this purpose, animals were infected with *P. berghei* ANKA parasites and four days after infection, animals were treated with suboptimal doses of AV (0.1 mg·kg^−1^, intraperitoneally) [29], 4‐NB (88 mg/kg, intraperitoneally) [28] or a combination of both (0.1 mg·kg^−1^ AV plus 88 mg·kg^−1^ 4‐NB, both administered intraperitoneally). Compounds were administered every 24 h for 5 days, and a control group of untreated animals was also performed (Fig. 4 and Supporting Information Fig. S2). Compared to untreated animals, mice treated with AV alone exhibited a slight reduction in parasitemia levels (Fig. 4A) and experienced significantly extended survival (Fig. 4B). However, animals treated with AV in combination with 4‐NB experienced a significantly more pronounced decrease in parasitemia, followed by a slower increase in parasitemia levels and extended survival time compared to those animals that received only AV. Mice treated with 4‐NB alone showed no reduction in parasitemia levels but a slightly reduced survival compared to untreated animals. These results indicate that 4‐NB possesses no antimalarial activity by itself but enhances the efficacy of AV *in vivo*.

## Discussion

AV is a current treatment for malaria. Its mechanism relies on competing with UQH_2_ for binding at cytochrome bc1 [10]. Its toxic effects on the parasite are thus due to inhibition of UQ‐redox regeneration, a process required for the activity of the DHODH enzyme involved in pyrimidine biosynthesis. However, parasites have developed resistance to AV and its combination therapy with proguanil [2, 10]. Our group previously found that malaria parasites can be protected from AV toxicity by synthetic UQ analogs, suggesting that, conversely, decreasing UQ biosynthesis could improve AV efficacy [6]. Exploring this possibility, we identified 4‐NB as an inhibitor of UQ biosynthesis with poor antimalarial potency on its own (IC_50_ > 2 mM at 48 h) but which strongly potentiated AV activity *in vitro* [6]. In this article, we questioned whether the molecular target of 4‐NB could be the 4‐HPT and if its AV potentiation effect could be exploitable for *in vivo* use.

Indeed, 4‐NB inhibited the 4‐HPT enzymatic activity in mutant strains of *S. cerevisiae* complemented with *PfCOQ2* in a competitive manner with the aromatic substrate. It should be noted, however, that while 4‐NB can inhibit UQ biosynthesis and 4‐HPT in malaria parasites, it should not be considered a specific tool for studying the effects of UQ depletion. Previous work showed that 4‐NB may also affect other mitochondrial processes [6]. Even so, 4‐NB effects on UQ metabolism seem to be a rational explanation for the observed potentiation of AV while preserving proguanil‐AV synergy. Importantly, this preservation of the synergistic effect strongly suggests that the mechanism through which proguanil potentiates AV—despite still not being well understood—is different from the mechanism by which 4‐NB achieves the same effect.

Besides *Plasmodium* parasites, 4‐NB was shown to interfere with UQ biosynthesis in highly diverse organisms, such as animal cells, cyanobacteria, and bacteria [34, 38, 39, 40]. In human fibroblasts, the compound efficiently inhibited UQ biosynthesis, but its cytotoxicity only became evident after incubating the cells for several days at concentrations around 3–4 mM [38, 39]. This low toxicity of the compound is not surprising, considering that several cell types can sustain ATP production through glycolysis. Furthermore, in certain animal and plant tissues, UQ has a prolonged half‐life of up to 100 h [41, 42].

In line with its low cytotoxicity *in vitro*, *in vivo* studies have confirmed that 4‐NB exhibits toxic effects only at relatively high doses. For example, within 24 h after intraperitoneal injection, the lethal dose 50 (LD50) values for 4‐NB were 1210 mg·kg^−1^ in rats and 880 mg·kg^−1^ in mice [28]. Following oral administration, the LD50 was 1960 mg·kg^−1^ in rats and 1470 mg·kg^−1^ in mice [28, 43]. However, further studies showed that a significant portion of orally administered 4‐NB is metabolized by gut microbiota into other compounds in marmosets [44]. Signs of oral toxicity included increased irritability, aggressiveness, convulsions, hind limb paralysis, exhaustion, rapid breathing, and purulent bloody lacrimation. Histopathological findings included erythrocyte infiltration in the liver and myeloid metaplasia in the red pulp of the spleen [28]. The toxic mechanism of 4‐nitrobenzoate is believed to be related to the oxidation of hemoglobin into methemoglobin, as demonstrated in studies using rat liver [45]. Mechanistically, 4‐nitrobenzoic acid induces methemoglobinemia due to the reduction of its nitro group, which oxidizes hemoglobin into methemoglobin. This effect was demonstrated in multiple *in vivo* studies and is particularly relevant because the redox imbalance also triggers splenic changes, such as hemosiderin deposition and congestion [46]. In any case, as noted, toxicity does not appear to occur at low doses, supporting the notion that this compound remains a promising starting point for the further evaluation of structurally related molecules with therapeutic potential. Altogether, the evidence positions 4‐NB as a chemically tractable scaffold with favorable toxicological margins for future drug development efforts.

In Plasmodium, the effects of 4‐NB were also found to be time‐dependent, with more pronounced antiparasitic activity observed after prolonged incubation [6]. Given its ability to inhibit mammalian 4‐HPT enzymes and interfere with ubiquinone biosynthesis [38], we next sought to investigate the impact of 4‐NB in our experimental context, particularly when combined with AV.

The presence of 0.5 mM 4‐NB did not significantly reduce the CC_50_ value of AV in the LLC‐MK2 cell line, reinforcing the interpretation that 4‐NB could be used to potentiate AV antimalarial selectivity against animal cells. Furthermore, our experiments using AV plus 4‐NB in combination in a murine malaria model were consistent with the *in vitro* results and 4‐NB improved the effects of administering suboptimal doses of AV in mice. At the same time, 4‐NB *per se* did not show any antimalarial effects but was well tolerated by animals. In addition to the potential combinatorial use of 4‐NB and AV, these findings highlighted the importance of UQ biosynthesis for malaria parasites and suggested that *PfCOQ2* could be a therapeutic target to enhance the efficacy of AV. In that respect, 4‐NB could be a starting point for designing molecules capable of boosting the efficacy of AV or AV plus proguanil combinations for malaria treatment.

As mentioned, in this study, we focused on 4‐NB because it had been previously tested in *Plasmodium* and has become the reference tool for studying the effects of UQ depletion. Indeed, 4‐NB has already demonstrated its ability to interfere with UQ biosynthesis in highly diverse organisms such as animal cells, cyanobacteria, and bacteria [34, 38, 39, 40]. In human fibroblasts, the compound efficiently inhibited UQ biosynthesis, but its cytotoxicity only became evident after incubating the cells for several days at concentrations around 3–4 mM [39]. This low toxicity of the compound is not surprising, considering that several cell types can sustain ATP production through glycolysis. Furthermore, in certain animal and plant tissues, UQ has a prolonged half‐life of up to 100 h [41, 42]. In *Plasmodium*, the effects of 4‐NB are also time‐dependent [6].

Beyond 4‐NB, other authors have tested similar compounds as tools to inhibit 4‐HPT activity in other organisms [34]. Evidence suggesting that 4‐HB analogs act on 4‐HPT activity in some model organisms includes the following: (i) inhibitory effects on UQ formation and (ii) reversal of toxic effects and/or UQ biosynthesis inhibition by the addition of exogenous 4‐HB, UQ, or dUQ [34, 39]. Only Alam et al. in 1975 [47] and Nowicka et al. in 2016 [40] demonstrated that 4‐HB analogs directly affect 4‐HPT enzymatic activity in mitochondrial preparations from animal tissues and cyanobacteria, respectively. Nowicka et al. only tested 4‐NB, while Alam et al. [47] tested acetyl salicylate, 5‐methyl salicylate, 5‐methoxy salicylate, 4‐chlorobenzoate (4‐ClB), pABA, 4‐sulfamoylbenzoate (carzenide), 4‐hydroxymercuribenzoate, procaine (2‐diethylaminoethyl 4‐aminobenzoate), and some catecholamines identified as potential regulators of 4‐HPT activity: serotonin, dopamine, and norepinephrine. Nowicka et al. [40] showed that 4‐NB inhibited the formation of prenylated products from [^14^C] 4‐HB, while Alam et al. demonstrated this inhibition using compounds with modifications in the C4 substituent of 4‐HB. Among these compounds, pABA also reduced the formation of prenylated products from [^14^C] 4‐HB, which led Alam et al. [47] to further investigate this phenomenon. The authors demonstrated that, similar to 4‐HB, pABA can also be prenylated by polyprenyltransferases in animal tissues. However, the resulting product, 3‐polyprenyl‐pABA, cannot be deaminated to continue in UQ biosynthesis. In contrast, yeasts can form UQs from pABA because, in addition to prenylating pABA, they can also deaminate 3‐polyprenyl‐pABA [48].

Considering the above, the literature concludes that, for the 4‐HPT enzyme to prenylated a given aromatic molecule, the substrate must meet two requirements: the presence of a substituent in the C4 position with the ability to transfer electrons (‐OH, ‐NH_2_, among others) and a strong electron‐attracting group in the C1 position (the carboxyl group) to generate sufficient electron density at the C3 position [34]. This electron density allows for a nucleophilic attack on the oxygen–carbon bond of the polyprenyl pyrophosphate. However, the size and electronegativity of radicals in the C4 position may also reduce nucleophilic interaction at the C3 position, making alkylation unfeasible. In line with this reasoning, this study observed pharmacological potentiation phenomena of AV using methyl 4‐HB, 2‐methyl 4‐HB, and all 4‐HB analogs with modifications in the C4 radical, except for carzenide. The compounds showing the most significant potentiation effects on AV activity were 4‐NB, 4‐ClB, and 4‐BrB, which remarked the importance of an electron‐drawing residue at C4. No potentiation phenomena were observed for any antimalarial when using sulfanilamide (control), and only a reduction of approximately 30% in Proguanil's IC_50_ value was achieved with methyl 4‐HB. These results further suggest that the mitochondrial target of Proguanil is likely not directly related to UQ metabolism. We also concluded that, overall, the most significant potentiation effects on AV activity are achieved by 4‐HB analogs with simple modifications (‐Br, ‐Cl, ‐NO_2_) to the substituent in the C4 position. Additionally, methyl 4‐HB and 2‐methyl 4‐HB also showed some potential to enhance AV's effects. A review of the literature revealed that two compounds in the collection had been previously tested in parasites: PAS [35] and sulfanilamide [36]. No evidence for a UQ‐related mechanism has been reported for any of them. Sulfanilamide is a known folate biosynthesis inhibitor, while PAS has been described as a potent inhibitor of exogenous pABA transport (IC_50_ for transport of approximately 200 nm) [35]. In agreement with this, we found no potentiation effect of those two compounds on AV action. Interestingly, a slight potentiation effect on AV activity was also observed using pABA. It is important to note that pABA is a metabolite produced by the parasite and is present in human blood [49]. Taylor in 1957 [50] showed that supplementing the diet of chicks infected with *P. gallinaceum* with high doses of pABA reduced parasitemia levels. However, this antimalarial effect of pABA could be reversed by adding 4‐HB to the diet, indicating that pABA may interrupt UQ biosynthesis.

As far as we know, this is the first time that ubiquinone biosynthesis has been targeted for the treatment of an infectious disease, with supporting evidence from animal models. This is particularly relevant given that several pathogens synthesize UQ and are also sensitive to AV. For example, AV and similar analogs are already used in the treatment of other diseases, such as babesiosis [51] and toxoplasmosis [52], and are being investigated for the treatment of leishmaniasis [53], among other infectious diseases. Therefore, the conclusions and strategy used in this work to enhance AV efficacy may also be of interest to many other diseases.

## Conclusions

Our study showed that 4‐NB effectively inhibited 4‐HPT in *Plasmodium falciparum*, this being the likely reason for the previously observed decrease UQ biosynthesis in malaria parasites. This inhibition not only enhanced the antiplasmodial efficacy of AV but also increased its selectivity compared to animal cells, without detrimental effects on proguanil efficacy. Furthermore, the combination of 4‐NB and AV significantly improved therapeutic outcomes in a murine malaria model, remarking the potential of 4‐HB analogs as an adjuvant to current antimalarial therapies. Finally, testing various 4‐HB analogs allowed us to confirm some of the chemical requirements for these compounds to potentiate AV activity. These findings underscored the importance of targeting UQ biosynthesis in malaria parasites and may provide a promising strategy to overcome resistance and enhance the efficacy of existing antimalarial drugs.

## Author contributions

IBV, MC, GOC, MFS, MAP, and MMF contributed to conceptualization, formal analysis, investigation, methodology, and writing. AMK, AH, and MC also contributed to project administration, funding acquisition, supervision, and writing – review and editing.

## Conflict of interest

The authors IBV, MC, and AMK have disclosed the use of several chemical analogs of 4‐hydroxybenzoate analogs in combination with AV and/or proguanil for the treatment of parasitic infections in the Brazilian patent application number BR 102021 006559 1. Besides this, all the authors declare no other conflict of interest.

## Supporting information


**Fig. S1.** PfCOQ2 complementation and enzymatic activity.
**Fig. S2.** 4‐Nitrobenzoate improves the efficacy of atovaquone *in vivo*.

## Data Availability

The data that support the findings of this study are available from the corresponding author marcell.crispim@unifal-mg.edu.br upon reasonable request. The supporting document file includes additional replicates of the experiments shown in Figs 3 and 4 of the main manuscript.
